# IoT Adaptive Dynamic Blockchain Networking Method Based on Discrete Heartbeat Signals

**DOI:** 10.3390/s20226503

**Published:** 2020-11-14

**Authors:** Xueyang Hu, Yili Zheng, Yu Su, Rui Guo

**Affiliations:** 1 School of Technology, Beijing Forestry University, Beijing 100083, China; huxueyang@bjfu.edu.cn; 2 Chaintofuture Technology Limited, Beijing 100081, China; suyu@ultrain.io (Y.S.); rayguo@ultrain.io (R.G.)

**Keywords:** blockchain, IoT, adaptive dynamic networking, BFT consensus algorithm, heartbeat monitoring

## Abstract

The combination of blockchain technology and Internet of Things (IoT) technology has brought many significant advantages and new development directions. With the development of embedded technology and 5G communication technology, the performance limitations and network limitations that are traditionally believed to restrict the application of blockchain technology to IoT devices have been broken. The development of “blockchain + 5G + IoT” provides reliable data from the source for the blockchain, linking the credible mapping of physical assets and digital assets. However, at the beginning of the blockchain design, the application of the IoT was not fully considered, so there have been some obvious defects in applying the blockchain technology in the IoT. In the Byzantine fault tolerance (BFT) consensus algorithm of traditional blockchain, the entire blockchain network will become paralyzed when more than 1/3 of the nodes in the network are offline. However, in IoT applications, this situation is likely to occur and greatly limits the security and stability of the application of blockchain technology in the IoT. In order to solve this problem, we proposed an IoT adaptive dynamic blockchain networking method based on discrete heartbeat signals. The feature of the method is to set a different monitoring time for each group of nodes, that is, discrete heartbeat signals monitoring. When the number of nodes gradually decreases, the IoT adaptive dynamic blockchain network can dynamically adapt to this process. Even when more than 1/3 of the IoT are offline, the adaptive dynamic IoT blockchain network can maintain stable running. This method also has the advantages of a short network expectation recovery time and avoids instantaneous system paralysis caused by the thundering herd effect. This research improves the security and stability of the application of blockchain technology in the IoT, and provides the necessary technical foundation for the better combination of blockchain technology and IoT technology.

## 1. Introduction

**Figure 1:**
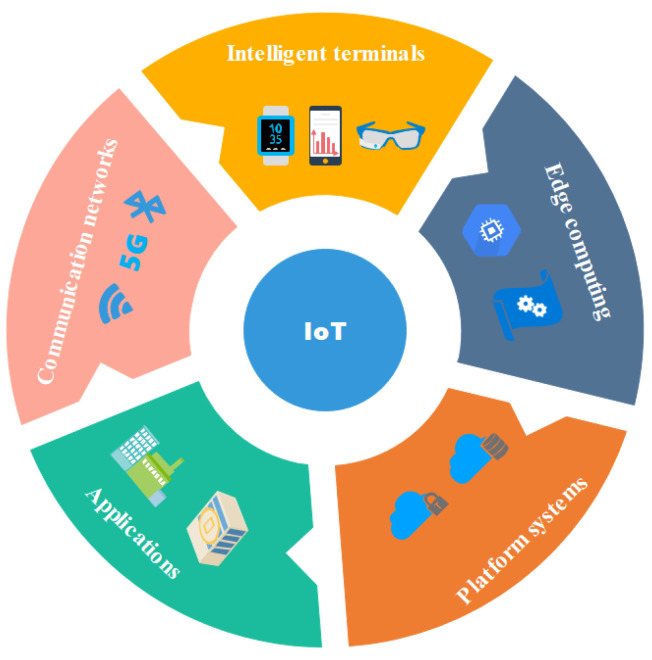
Structure of the Internet of Things (IoT) ecosystem.

The Internet of Things (IoT) is an environment in which physical things are embedded with electronics, software, sensors, and network connectivity, with the ability to transfer data and generate collaborative outcomes [1]. The IoT has gradually developed into a mature ecosystem composed of intelligent terminals, communication networks, edge computing, platform systems, and IoT applications, as shown in Figure 1 [2,3].

Blockchain refers to a type of data structure that enables the identification and tracking of transactions digitally and the sharing of this information across a distributed network of computers, creating a distributed trust network [4]. The distributed ledger technology offered by blockchain provides a transparent and secure means for tracking the ownership and transfer of assets. Blockchain technology offers a way for untrusted parties to reach a consensus on common digital records [5].

With the development of embedded technology and 5G communication technology, the performance limitations and network limitations that are traditionally believed to restrict the application of blockchain technology to IoT devices have been broken.

In the era of digital economy, the combination of blockchain and IoT will further promote the credible securitization of physical assets. The development of “blockchain + 5G + IoT” provides reliable data from the source for the blockchain, linking the credible mapping of physical assets and digital assets.

### 1.1. Motivation

The combination of IoT and blockchain improves the security, credibility, traceability, and liquidity of distributed data, and realizes the reliability of operations between IoT devices and the reliability value flow between different IoT devices. Through the combination with blockchain technology, the IoT has many new advantages [6]. The IoT built with blockchain technology does not need intermediate servers, avoiding expensive operation and maintenance costs. The verification and consensus mechanism of the blockchain helps prevent illegal or malicious nodes from accessing the IoT [7,8]. All data transmitted on the blockchain are strictly encrypted, and user data and privacy will be more secure. The distributed peer-to-peer (P2P) structure and transparent algorithm of the blockchain can establish a low-cost trust, and promote the horizontal flow of information across the main body and multi-party collaboration [9]. At the same time, as long as the data are written into the blockchain consensus, it is difficult to modify, and it can also rely on the chain structure to trace the source.

In recent years, the combination of blockchain technology and the IoT has been applied to industrial, medical care, supply chain, environmental protection, energy, agriculture, and other fields. In 2015, Microsoft launched the Azure blockchain as a service (Baas) based on Ethereum. The Azure applies blockchain technology to provide a cloud-based one-click blockchain development environment [10]. In 2018, the global blockchain business application leader, Ultrain, cooperated with a new energy company to build a carbon emissions trading platform [11]. In 2019, IBM released a new blockchain network for optimizing supply chain information management. Nokia, Cisco, Vodafone, and other technology companies participated in this blockchain [12]. The combination of blockchain technology and IoT technology has brought many significant advantages and new development directions.

### 1.2. Current Issues

However, at the beginning of the blockchain design, the application of the IoT was not fully considered, so there have been some obvious defects in applying the blockchain technology in the IoT. In the Byzantine fault tolerance (BFT) consensus algorithm of traditional blockchain, the entire blockchain network will become paralyzed when more than 1/3 of the nodes in the network are offline [13]. However, in IoT applications, this situation is likely to occur and greatly limits the security and stability of the application of blockchain technology in the IoT.

For example, a distributed energy measurement and transaction blockchain network composed of 1000 IoT devices is where energy tokens are issued, circulated, exchanged, and consumed in the blockchain network [14]. Due to the IoT devices being controlled by the terminal, the operating state of each IoT device may be different. Some IoT devices may only be turned on during working hours, and will be turned off during the rest of the time. In addition, the working time of each terminal is also different, so the time of turning on and off the IoT device will also be different. At the same time, the network conditions will also fluctuate because the IoT devices are running on the public network, which will suddenly cause some of the IoT devices to disconnect from the network. Therefore, we are faced with a dynamic IoT network. In this network, there are IoT devices joining and exiting at any time, and the number of online IoT devices is not fixed. When the IoT nodes go offline, the BFT consensus algorithm has a certain probability of becoming unable to produce valid blocks, which leads to empty blocks in the current round of accounting. When more than 1/3 of the IoT nodes go offline, it will lead to the failure to complete the BFT consensus algorithm, and the system will be stuck in the current round and be unable to continue, which will paralyze the entire blockchain network. This problem greatly limits the security and stability of the application of blockchain technology in the IoT.

### 1.3. Research Contribution

In order to solve the problem of the entire blockchain network becoming be paralyzed when more than 1/3 of the IoT devices in the BFT consensus algorithm blockchain network are offline, we proposed an IoT adaptive dynamic blockchain networking method based on discrete heartbeat signals. The feature of the method sets a separate monitoring time for each monitored node, that is, discrete heartbeat signals monitoring. When the number of nodes in the blockchain network gradually decreases, the IoT adaptive dynamic blockchain network can dynamically adapt to this process. After the disconnected nodes come back online, the IoT adaptive dynamic blockchain network will also automatically connect these nodes to the network and let them continue to provide services.

The contribution of this research is to propose a technical solution for the IoT adaptive dynamic blockchain networking method based on discrete heartbeat signals. It realizes the dynamic adaptation of the IoT blockchain network. Even when more than 1/3 of the IoT are offline, the adaptive dynamic IoT blockchain network can maintain stable running. Compared with fixed-period heartbeat monitoring, this method also has the advantages of a short expected recovery time of the network and avoids instantaneous system paralysis caused by the thundering herd effect. This research improves the security and stability of the application of blockchain technology in the IoT, and provides the necessary technical foundation for the better combination of blockchain technology and IoT technology.

The rest of the paper is organized as follows. In Section 2, the background and the related work are presented, and in Section 3, we describe our main contribution, which is an IoT adaptive dynamic blockchain networking method based on discrete heartbeat signals. Next, we present the performance verification in Section 4 and the conclusion in Section 5.

## 2. Background and Related Work

### 2.1. Important Definitions of Blockchain

To understand more about blockchain, its underlying technology, and our research, here are some important definitions. The following definitions refer to the Oracle blockchain platform [15].

Decentralized trust: The key reason that organizations use blockchain technology, instead of other data stores, is to provide a guarantee of data integrity without relying on a central authority. This is called decentralized trust through reliable data.

Blockchain blocks: The name blockchain comes from the fact that the data are stored in blocks, and each block is connected to the previous block, making a chainlike structure. With blockchain technology, you can only add (append) new blocks to a blockchain. You cannot modify or delete any block after it gets added to the blockchain.

Consensus algorithms: Algorithms that enforce the rules within a blockchain system. Once the participating parties set-up rules for the blockchain, the consensus algorithm ensures that those rules are followed.

Blockchain nodes: Blockchain blocks of data are stored on nodes—the storage units that keep the data in sync or up to date. Any node can quickly determine if any block has changed since it was added. When a new, full node joins the blockchain network, it downloads a copy of all the blocks currently on the chain. After the new node synchronizes with the other nodes and has the latest blockchain version, it can receive any new blocks, just like the other nodes.

### 2.2. Distributed System and Heartbeat Monitoring

A distributed system is a system composed of a group of computer nodes that communicate through the network and coordinate work in order to accomplish tasks [16]. The distributed system uses cheap, ordinary machines to complete the calculation and storage tasks that cannot be completed by a single computer. The purpose is to use more machines to process more data.

Heartbeat monitoring is a technology used in distributed systems to remove abnormal nodes in real time to avoid assigning tasks to abnormal machines [17]. Heartbeat refers to the way that the machine reports the current node status to other nodes at a fixed frequency. The heartbeat monitoring method sets a timeout period, and the device is considered abnormal if no heartbeat signal is received within this time. Heartbeat monitoring is used to detect faulty nodes in distributed systems and improve system security.

### 2.3. BFT Consensus Algorithm

BFT technology is a type of fault tolerance technology in the field of distributed computing. BFT technology comes from the Byzantine Generals Problem, first proposed by Turing Award winner Lamport et al. in 1982 [18]. The Byzantine Generals problem is a well-known example to describe distributed consensus. The Byzantine assumption is a model of the real world. Due to hardware errors, network congestion or interruption, and malicious attacks, computers and networks may show unpredictable behaviors [19]. BFT technology is designed to deal with these abnormal behaviors and meet the requirements of the problem. It has been proven that the BFT algorithm’s fault tolerance rate is 1/3. Castro and Liskov proposed the practical Byzantine fault tolerance (PBFT) in 1999 [20]. This is an improvement to BFT that reduces the complexity of BFT and makes it available for practical use. The distributed trust of the blockchain and the Byzantine Generals problem are similar in some situations. Therefore, PBFT has become a consensus algorithm applied in the blockchain. The PBFT consensus algorithm achieves consensus by passing messages between nodes, which has the advantage of consensus certainty.

In recent years, BFT has been optimized for different applications, resulting in some derived consensus algorithms, such as Tendermint BFT (TBFT) [21], Redundant-BFT (RBFT) [22], and Delegated proof of stake BFT (DPos + BFT) [23]. In 2018, Feng and others proposed the Scalable Dynamic Multi-Agent Practical Byzantine Fault-Tolerant Consensus in Permissioned Blockchain [24]. This approach enhances the scalability. However, the above consensus algorithms can only tolerate faults less than 1/3. It should be noted that the BFT mentioned in other sections in this research does not only refer to the BFT consensus algorithm, but includes other consensus algorithms derived from BFT.

### 2.4. Related Work

There are currently two main solutions for the problem where the blockchain network will become paralyzed when more than 1/3 of the nodes in the network are offline in the BFT consensus algorithm.

The first solution is to accept and recognize the network paralysis. This acceptance and recognition of network paralysis is not a manifestation of inaction, but a choice based on the traditional application scenarios of the BFT consensus algorithm. The traditional application scenarios of the BFT consensus algorithm is generally not used in large-scale blockchain networks, which means that there are very few nodes in the network. These nodes are deployed in the data center and directly connected with the optical fiber network. In this case, the node will hardly fail due to fluctuations in the network or power supply. Therefore, the probability of failure of more than 1/3 of the nodes is very low [25]. Once it happens, there is a great possibility of malicious attacks. The network paralysis that occurs at this time will cause the blockchain to stop producing blocks and stop running. This paralysis protects the blockchain network from operating in a risky state and prevents the spread of risks. However, this acceptance and recognition of network paralysis does not apply to the application of the Internet of Things, as IoT devices will frequently go offline in the blockchain network due to network fluctuations or owner operations. This kind of network paralysis caused by expected reasons cannot be accepted and recognized by the IoT blockchain. We need a blockchain network that can run a large number of IoT nodes and also run stably when a large number of nodes are offline.

The second solution is to use the large-scale node blockchain consensus algorithm Proof of Work (PoW). In 1999, Markus Jakobsson and Ari Juels introduced the PoW concept to resist DDOS attacks and anti-spam. In 2008, Satoshi Nakamoto adopted the PoW method in Bitcoin systems, and this made the PoW method popular [26]. In the PoW consensus algorithm, the blockchain concept of mining is introduced as a proof of work. The PoW consensus algorithm guarantees the safe running of large-scale node blockchain networks through the mining mechanism, and allows arbitrary entry and exit of nodes. This solution solves the problem of network paralysis when more than 1/3 nodes of the traditional BFT consensus algorithm are offline, but the mechanism of PoW mining is not suitable for the IoT network. The mechanism of PoW mining wastes a lot of the computing power of nodes, making the work efficiency of the network very low [27]. For IoT nodes, the computing power is relatively limited, and we need to apply the computing power to actual work scenarios without mining. Proof of Stake (PoW) and Proof of Assets (PoA) are two consensus algorithms proposed to reduce the waste of computing power caused by mining, but to a certain extent, they sacrifice the decentralization of the consensus algorithm [28]. In particular, PoA relies on preset authorized nodes (signers) to be responsible for generating blocks, so it has a high degree of centralization [29]. Obviously, these two consensus algorithms are not suitable for IoT blockchain networks with equal node rights.

In summary, the above two solutions cannot satisfactorily solve the problems in the IoT blockchain, so our research is very meaningful.

## 3. Proposed IoT Adaptive Dynamic Blockchain Networking Method

In order to analyze the above problem, we assume a BFT consensus protocol blockchain network composed of IoT devices. The IoT devices in the blockchain in this research refer to embedded system devices running an operating system. The embedded system device usually runs the Android or Linux operating system and realizes the IoT function through the sensor module and the network module.

The total number of IoT nodes in the blockchain network is m, and the blockchain uses the BFT consensus algorithm. Under normal circumstances, the blockchain will generate a data block in each block-out period t. When IoT devices become faulty nodes due to network failure or other reasons, the security and stability of the blockchain network will be threatened. Supposing there are h abnormal IoT nodes at a certain moment, according to the relationship between the number of faulty nodes h and the total number of nodes m, the analysis will be divided into two cases.

Case 1: 0 < h < 1/3m.

In this case, part of the IoT nodes are offline, but the number of faulty nodes h is less than one-third of the total number of nodes m. At this time, the blockchain network has a certain probability to select the faulty node as the accounting node, which will cause the blockchain to be unable to produce valid blocks. The empty blocks in the accounting process make the blockchain network risky.

Case 2: h≥1/3m.

In this case, the number of faulty nodes h is greater than one-third of the total number of nodes m, which will lead to the failure of the BFT consensus algorithm. The blockchain network is stuck in the current round and cannot continue, resulting in the entire paralysis. h = 1/3m is an absolute threshold. Once the number of offline nodes is greater than or equal to 1/3m, the BFT consensus algorithm will not be achievable.

It needs to be pointed out that although the BFT consensus algorithm can still be achieved when the offline nodes are less than 1/3m, when close to 1/3m nodes are offline, the system will be in a high-risk state. Due to any kind of accidental event, the number of offline nodes of the system reaching the 1/3m threshold will cause the consensus algorithm to fail being reached. Therefore, this high-risk state close to 1/3m is also undesirable.

### 3.1. Fixed-Period Heartbeat Monitoring

In order to solve the security and stability problems caused by the high proportion of faulty nodes in the blockchain network, we need to check the status of the IoT nodes in the network and remove the faulty nodes in real time. Therefore, the solution we proposed is to deploy a smart contract similar to the heartbeat monitoring of the distributed system in the blockchain network, named fixed-period heartbeat monitoring.

The task of fixed-period heartbeat monitoring is to enable the normal and effective IoT nodes in the network to actively send a message to the blockchain network to prove their existence. The content of the fixed-period heartbeat monitoring smart contract is as follows.

Step 1: All valid IoT nodes in the blockchain must send the check-in transaction in the smart contract within the specified period T to complete the check-in on the chain.

Step 2: Check nodes that have not sent heartbeat messages in time, and remove the unregistered IoT nodes from the blockchain network.

Fixed-period heartbeat monitoring seems to solve the problem very well, but it has the two following disadvantages in practical applications:

Disadvantage 1: Security risks caused by the thundering herd effect.

The fixed-period heartbeat monitoring has a fixed monitoring period. When the time is up, it checks nodes that have not sent heartbeat messages in time. If the faulty nodes exceed the system threshold at a certain time, excessive threads will compete for system resources and lead to the fixed-period heartbeat monitoring system to be bypassed, causing a catastrophic impact on the system. The probability of this happening is proportional to the monitoring period T. This kind of instantaneous system paralysis caused by all threads competing for system resources at a certain time is called the thundering herd effect. The thundering herd effect greatly threatens the security of the block network.

Disadvantage 2: Low recovery performance caused by fixed period.

The fixed-period heartbeat monitoring can only monitor and remove the IoT node at a fixed time point. Therefore, the network recovery time is limited by the monitoring period T, and cannot be adjusted adaptively as the state of the IoT node changes in the network.

Based on the analysis of the fixed-period heartbeat monitoring, we found that this method cannot complete the task under the requirements of high security and high recovery performance. Therefore, we need to find a better solution.

### 3.2. IoT Adaptive Dynamic Blockchain Networking Method Based on Discrete Heartbeat Signals

In order to prevent the thundering herd effect and shorten the network recovery time, we propose an IoT adaptive dynamic blockchain networking method based on discrete heartbeat signals. The most effective way to avoid the thundering herd effect is to split the node detection work. Therefore, the adaptive dynamic blockchain networking method sets a different monitoring time for each group of nodes, samples nodes for checking, and removes the faulty nodes, that is, discrete heartbeat signals monitoring.

Reasonable node grouping is the key point of this method. Therefore, we used artificial intelligence (AI) algorithms in the node grouping to ensure the rationality, that is, the K-Means clustering algorithm. The K-Means algorithm is a data mining technology widely used in science and industry [30]. Its advantage is that the calculation process is simple and it can process a large amount of data in a short time [31].

The algorithm of IoT adaptive dynamic blockchain networking based on discrete heartbeat signals is divided into two parts; one is sampling IoT nodes to check and the other is checking and removing the unregistered nodes as follows:

Algorithm Part 1: Divide nodes clusters.

Step 1: Divide the fixed-period T into n time nodes, and name the time node “checkpoint.” The interval of each checkpoint is T/n. n is determined by the degree of association δ. The value range of the δ is 0 to 1.

Step 2: In most applications, whether the IoT nodes in the blockchain can provide computing power is closely related to time, so we use the time zone division method to divide all nodes into 24 sets, which are marked as $C_{i}$. The value range of i is 0≤i≤23.

Step 3: If the set $C_{i}$ has no more than Min nodes, it is grouped into the left. Min is set as 3% of the total number of nodes m. For example, if the number of nodes in set $C_{i}$ is less than Min, then group them into set $C_{i-1}$.

Step 4: Delete the empty set generated after grouping to obtain the effective sets.

Step 5: Use a two-dimensional feature attribute (X,Y) to describe the node. X is the number of the time zone to which it belongs, and Y is the number of historical blocks produced by the node.

Step 6: Randomly select the $k_{i}^{}$ centroid of each valid set. $k_{i}^{}$ is the number of centroids in each valid set and determined by Equation (1). $N(C_{i})$ is the number of nodes in set $C_{i}$.

(1) $$k_{i}^{}=N(C_{i})/Min$$

Step 7: Node (X,Y) and $k_{i}^{}$ centroids use the K-Means clustering algorithm to find K node clusters. K is the sum of $k_{i}^{}$. According to the specific network characteristics, the weights of X and Y in the K-Means clustering algorithm can be set. By default, the X weight and the Y weight are also 50%.

The K-Means clustering algorithm first calculates the distance from each centroid to each node by Equation (2).

(2) $$D_{k_{i}^{}}(X,Y)=\sqrt{{\left(X-X_{k_{i}^{}}^{}\right)}^{2}+{\left(Y-Y_{k_{i}^{}}^{}\right)}^{2}}$$

Secondly, divide the node into the nearest centroid to form node clusters.

Thirdly, calculate the new centroid $K_{i}$ of each cluster by Equation (3). $X_{i}$ and $Y_{i}$ refer to the coordinate value of the ith node belonging to the $K_{i}$ cluster.

(3) $$K_{i}(X,Y)=(\frac{\sum X_{i}}{n},\frac{\sum Y_{i}}{n})$$

Finally, iterate three times to complete the division of the node clusters [32].

Algorithm Part 2: Check and remove the unregistered nodes.

Step 1: After the division of the node clusters, a node is randomly sampled from each node cluster to form a checking set for this checkpoint.

Step 2: Nodes in the checking set will be checked and the node that is not registered will be removed from the valid network nodes.

Step 3: For the set of faulty nodes, the sampling weight α will be increased at the next checkpoint.

Step 4: Perform the above checking and removing steps in each checkpoint.

When h faulty nodes are detected by discrete heartbeat signals and removed, the number of effective nodes becomes m′, as shown in Equation (4).

(4) $$m^{\prime }=m-h$$

Each round of consensus will only select valid nodes as block producers, which can effectively avoid faulty nodes and improve the stability and security of the blockchain network.

The discrete heartbeat signals are based on the BFT consensus algorithm, which retains the consensus protocol and advantages of the BFT consensus algorithm. When the BFT consensus network runs the IoT adaptive dynamic blockchain networking method based on discrete heartbeat signals, the checking set node will send its heartbeat to the blockchain network with a specified transaction message. The BFT consensus algorithm will select the block producer node of the current checkpoint. The block producer will package the received heartbeat information and send this data block to all nodes in the network through the whole network broadcast. The node that receives the data block will compare the received heartbeat information with the heartbeat information in the data block. The judgment of the node status is completed under the support of the BFT consensus protocol. Finally, when the current round of consensus is completed and the block is produced, the network will execute the judgment result of the node status removing the faulty node.

In some of the following sections, we refer to the IoT adaptive dynamic blockchain networking method based on discrete heartbeat signals as discrete heartbeat monitoring for short.

**Figure 2:**
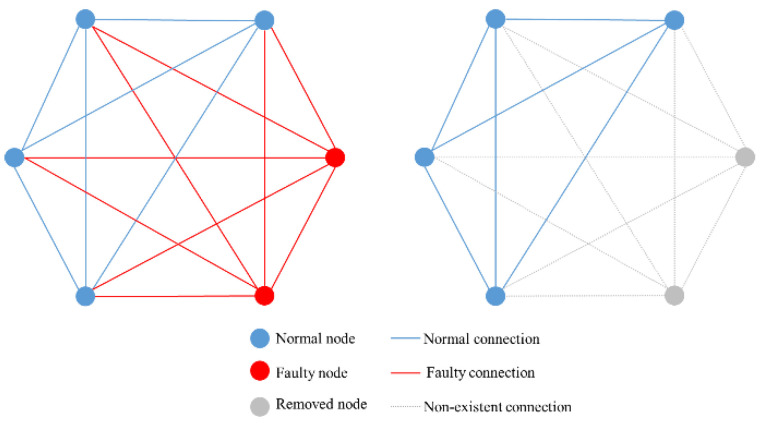
Network fault topology diagram. The heartbeat monitoring contract is not deployed on the left. The discrete heartbeat monitoring contract is deployed on the right.

Two network fault topology diagrams are shown in Figure 2. There are two faulty nodes in both figures. The network on the left does not deploy any heartbeat contracts. The faulty node will always exist in the blockchain network, resulting in a large number of faulty connections. When the faulty nodes are greater than or equal to 1/3, the BFT consensus will not be reached. The network on the right deploys discrete heartbeat monitoring, which will remove the faulty node from the blockchain network, ensuring that there will be no faulty connections in the network, so that when the faulty node is greater than or equal to 1/3, the network can still maintain normal operation.

## 4. Performance Verification

In order to verify the performance of the IoT adaptive dynamic blockchain networking method based on discrete heartbeat signals, we tested the networking of physical IoT nodes and the large-scale node blockchain network risk model. A qualitative comparison of the networking of physical IoT nodes shows the high stability and security of the network where the heartbeat contract is deployed. Through the quantitative analysis of the large-scale node blockchain network risk model, the performance of adaptive networking based on discrete heartbeat monitoring and fixed-period heartbeat monitoring is compared, and an optimization method for the expected recovery time of performance indicators is given.

### 4.1. The Qualitative Performance Verification by the Physical IoT Node Networking

#### 4.1.1. Test Environment

**Figure 3:**
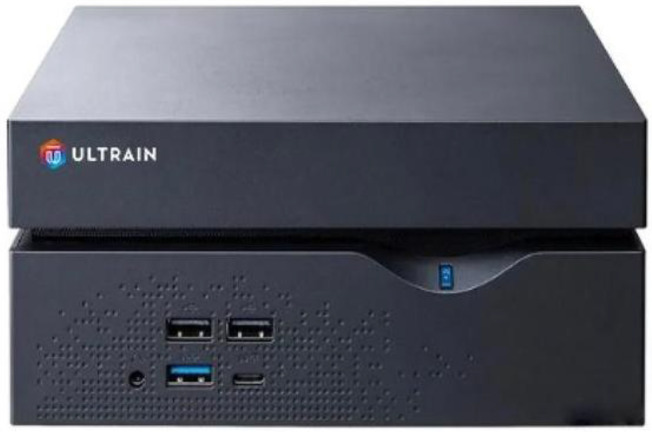
Pribox IoT node hardware.

In order to make the test more credible, we use Pribox as an IoT node in the Ultrain blockchain test network for testing. The Pribox IoT node hardware shown in Figure 3 is designed based on the RK3399 high-performance open-source platform, equipped with dual-core Cortex-A72 and quad-core Cortex-A53 processors, with a main frequency of up to 1.8 GHz. It is also equipped with an ARM Mail-T860 quad-core graphics processor, 4 GB of dual-channel DDR3 memory, with a network, audio and video, sensor, and other Internet of Things expansion interfaces, running the Ubuntu 16.04 operating system. The smart contracts used in the test are all developed by the Robin smart contract development framework.

In the Ultrain blockchain network system environment, the highest transactions per second (TPS) can reach 3000/s, but on different networks, especially sensor networks, the service time varies. Under the system, the consensus period is 10 s. When the business volume is below 3000/s, the average waiting time and average service time are both 10 s, which does not involve queuing theory. When the business volume is above 3000/s, under the assumption that the network is unobstructed, based on the Gossip protocol, every new transaction will be queued in the consensus node queue. Although the consensus node is randomly selected, the service is still the first-come first-served (FCFS), M/M/1 model.

The Ultrain blockchain system is based on the PBFT consensus algorithm and has a clear set of consensus nodes. Regardless of the additions or deletions made through consensus on the chain, the fault tolerance rate for consensus nodes is 1/3, and complete control needs to exceed 2/3, which has a great probability of avoiding Bribe Attack and Sybil Attack. We regularly publish the hashes of the consensus node collection in public, including, but not limited to, communities, company homepages, and other chains such as Ethereum, to prevent long-range attacks. No currency age is involved, and there is no Accumulation Attack problem. As for precomputing, we adopt a new random number generation algorithm based on the consensus node set, which can avoid this problem with great probability. Therefore, the network environment has high security.

#### 4.1.2. Network Availability Testing

We networked 36 pribox IoT nodes into a blockchain network of the BFT consensus algorithm. The fluctuation of the blockchain network is reproduced by means of offline nodes. In the test, we set that four devices will be offline every 5 min, and stop testing until more than 85% of the nodes are offline. It was tested in the following three network environments.

Environment 1: Do not deploy any heartbeat monitoring smart contracts.

Environment 2: Deploy a fixed-period heartbeat monitoring smart contract with a fixed period of 2 min.

Environment 3: Deploy an IoT adaptive dynamic blockchain networking method based on discrete heartbeat signals. The checkpoint interval is 20 s, each checkpoint detects six nodes, and the time to complete a round of all node detections is 2 min.

Judging the availability of the blockchain network by observing whether the blockchain network can reach the BFT consensus to complete the block production. The test results are shown in Table 1. “Y” means the network is available and “N” means the network is unavailable.

From the test results in Table 1, it can be seen that because environment 1 does not deploy any heartbeat monitoring algorithm, the blockchain network cannot exclude offline nodes. When the proportion of offline nodes is greater than or equal to one third, the BFT consensus algorithm cannot be completed. The network is unavailable.

Environment 2 and Environment 3 deploy the fixed-period heartbeat monitoring smart contract and discrete heartbeat monitoring smart contract, respectively. When the node is offline, the smart contract will remove the abnormal node from the consensus network so that the network can still remain available when more than one third of the nodes are offline.

Through the qualitative analysis of this test, we see that the two types of heartbeat monitoring smart contracts both show their effects after more than 1/3 of the nodes are offline, significantly improving the security and stability of the network.

Thus, this test did not reflect the performance difference between fixed heartbeat monitoring and discrete heartbeat monitoring. This is because in the quantitative test of physical test nodes, we can only visually observe the final block production results of the network, and cannot observe the changes in network nodes during the monitoring period. At the same time, as an extreme situation, the thundering herd effect will not appear when the number of nodes in the blockchain network is small. Due to the limitation of the number of physical node devices, it is difficult for us to use thousands of devices for this test, so the thundering herd effect cannot be reproduced in this test.

Based on the above problems, we have established a large-scale node blockchain network risk model for quantitative analysis to compare the performance difference between fixed-period heartbeat monitoring and discrete heartbeat monitoring.

### 4.2. The Quantitative Performance Verification by the Blockchain Network Model

#### 4.2.1. Definition of Blockchain Network Status Indicators

The change in blockchain network risk states can effectively demonstrate the performance of the adaptive dynamic blockchain networking method. The maximum fault tolerance of the BFT consensus algorithm is 1/3, which means that when the number of offline nodes exceeds 1/3 of the total number of nodes, the network will be paralyzed. Therefore, we define a paralyzed state when the proportion of offline nodes is between 33% and 100%. When the proportion of offline nodes is less than 33%, the network will not be paralyzed. However, as the proportion of offline nodes increases, the risk of network paralysis increases. We divide the range of 0 to 33% into four risk states based on the perspective of actual applications of network running and maintenance. In Table 2, five blockchain network states are defined according to the proportion of offline nodes in the network.

#### 4.2.2. Establishment of Large-Scale Node Blockchain Network Risk Model

In order to verify the performance difference between fixed-period heartbeat monitoring and discrete heartbeat monitoring, a large-scale node blockchain network risk model was established. This model is general and focuses on the probability and risk of node failure in the blockchain network.

In the large-scale node blockchain network risk model, assume that the total number of IoT nodes in the blockchain network is m. The nodes are independent of each other, and the probability of a node going offline is p=1/4. The probability of more than f=1/3 nodes failure P is very low, as shown in Equation (5).

(5) $$P=p^{\left(m\cdot f\right)}$$

From this perspective, the probability that the blockchain network is in a paralyzed state is very small. However, the nodes are only completely independent of each other in theory. In reality, many nodes are related. The network considers some habits of IoT device owners, such as some nodes being turned off at night, which greatly increases the instability of the blockchain network.

Therefore, to further simplify the model, the m nodes are divided into K clusters according to the K-Means clustering algorithm. The nodes in each cluster are related. The degree of association δ=1. Node clusters are independent of each other.

Suppose that at a certain time, multiple nodes in the system go offline, and the system enters a high-risk state and will become paralyzed at any time. The probability of producing empty blocks is close to 1/3, and the system is in a low availability state. At this time, the performance advantage of the IoT adaptive dynamic blockchain networking method based on discrete heartbeat signals is proved by comparing the time when the system is out of the high-risk state with the fixed-period heartbeat monitoring method. According to the above definition of the node cluster model, assuming that two node clusters are offline, the set of faulty nodes is named B.

#### 4.2.3. Parameter Setting and Performance Comparison

In order to show the performance of the system more intuitively, we introduce values into the model without loss of generality. Assume that m=96, |B|=32, and any node $b_{i}$ in B is offline. It should be pointed out that the above values are only general assumptions for the convenience of calculation and can be replaced with any reasonable values. We need to check more than five offline nodes and remove them from the blockchain network in order to move the system out of the high-risk state.

We divide the period T into m/K checkpoints. At the first checkpoint, one node is randomly selected from each node cluster obtained by the K-Means clustering algorithm to form a set H, where H∈m. If |H∩B|≥5, at the first checkpoint, more than five faulty nodes have been detected, moving the system out of the high-risk state. The probability P[(|H∩B|)≥5] of this event is shown in Equation (6). $C_{m}^{K}$ refers to the combination type of K nodes selected from the set m. $C_{B}^{i}$ refers to the combination type of i (0≤i≤4) nodes selected from the set B. $C_{m-B}^{K-i}$ refers to the combination type of (K−i) nodes selected from the set (m−B).

(6) $$P[(\left|H\cap B\right|)\geq 5]=1-\sum_{i=0}^{4}\frac{C_{B}^{i}\cdot C_{m-B}^{K-i}}{C_{m}^{K}}$$

The probability that more than five failed nodes can be detected before the nth-checkpoint (1≤n≤6) can be obtained by the recursive method, as shown in the first line of Table 3.

In the same situation, the fixed-period heartbeat monitoring’s period is T, so the system will leave the high-risk state at time T, but there will be no improvement within the time T, as shown in the second line of Table 3.

**Figure 4:**
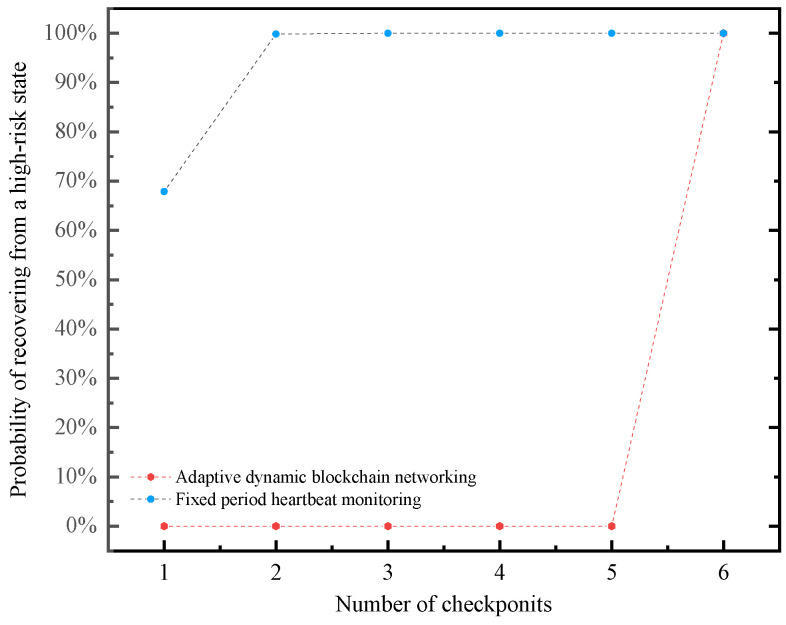
The performance comparison by the probability of recovering from a high-risk state.

The relationship between the probability of recovering from a high-risk state and the number of checkpoints can also be more intuitively represented by Figure 4.

It can be seen from the data analysis that the system has a 67.9% probability of recovering from a high-risk state at 1/6T, while the probability is more than 99.9% at 1/3T. The response time of adaptive dynamic blockchain networking is better than that of fixed-period heartbeat monitoring.

At the same time, we can also find that the impact of fixed-period heartbeat monitoring on the system is concentrated at *n* = 6, while the system impact of discrete heartbeat monitoring at each time is only 1/6 of the fixed-period heartbeat monitoring. The IoT adaptive dynamic blockchain networking method based on discrete heartbeat signals reduces the instantaneous impact on the system and avoids the thundering herd effect. Therefore, the IoT adaptive dynamic blockchain networking method is an excellent solution that meets the requirements of security and stability while having better performance.

#### 4.2.4. Optimization of Expected Recovery Time and Critical Discussion

The average time for the system recovering from a high-risk state is named the expected recovery time. The expected recovery time E(x) can be calculated by Equation (7). $x_{i}$ refers to the system recovery time. $p(x_{i})$ refers to the probability of system recovery at time $x_{i}$.

(7) $$E(x)=\sum_{i=1}^{n}[x_{i}p(x_{i})]$$

For example, in the above blockchain network model, the expected recovery time E(x) is calculated as shown in Equation (8). δ refers to a very small quantity.

(8) $$E(x)=\frac{1}{6}T\times 67.899\%+\frac{2}{6}T\times (99.838-67.899\%)+\delta \approx 0.22T$$

**Figure 5:**
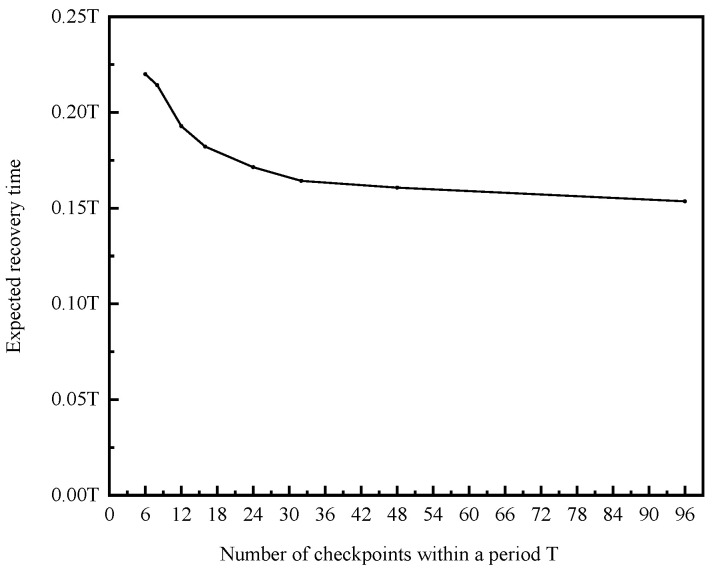
The relationship between the number of checkpoints within a period T and the expected recovery time.

The shorter expected recovery time indicates that the system can recover from a high-risk state in a shorter time when node fluctuations occur, which is an important indicator of system stability and security. We try to adjust some parameters in the adaptive dynamic networking to optimize the expected recovery time. Adjusting the number of checkpoints within a period T is an effective method, as shown in Figure 5.

It can be seen that the expected recovery time decreases as the checkpoints density increases. The safety and stability of the system can be optimized by setting more checkpoints.

However, setting too many checkpoints in a period will consume a lot of system resources and affect the normal working efficiency of the blockchain network. Therefore, it is necessary to set a reasonable number of checkpoints according to specific system performance and usage requirements. We recommend setting a reasonable number of checkpoints. This number is appropriate when the expected recovery time will not be significantly shortened with the number of checkpoints increasing.

## 5. Conclusions

The IoT adaptive dynamic blockchain networking method based on discrete heartbeat signals realizes the dynamic adaptation of the IoT blockchain network and the optimization of the BFT consensus algorithm. Even when more than 1/3 of the IoT are offline, the adaptive dynamic IoT blockchain network can maintain stable running. In the performance verification experiment, the expected recovery time is only 0.22T. The system has a 67.9% probability of recovering from a high-risk state at 1/6T, while the probability is more than 99.9% at 1/3T. This method also has the advantages of a short network expectation recovery time and avoids instantaneous system paralysis caused by the thundering herd effect. This research improves the security and stability of the application of blockchain technology in the IoT, and provides the necessary technical foundation for the better combination of blockchain technology and IoT technology.

## Figures and Tables

**Table 1:** Network availability testing results.

Proportion of Offline Nodes	0%	11.11%	22.22%	33.33%	44.44%	55.55%	66.66%	77.77%	88.88%
**Environment 1 availability**	Y	Y	Y	N	N	N	N	N	N
**Environment 2 availability**	Y	Y	Y	Y	Y	Y	Y	Y	Y
**Environment 3 availability**	Y	Y	Y	Y	Y	Y	Y	Y	Y

**Table 2:** The blockchain network states.

State	Low Risk State	Low to Medium Risk State	Medium Risk State	High Risk State	Paralyzed State
**Offline nodes proportion**	0–5%	5–15%	15–30%	30–33%	33–100%

**Table 3:** The performance comparison by the probability of recovering from a high-risk state.

*n*	1	2	3	4	5	6
**Adaptive dynamic blockchain networking**	67.899%	99.838%	>99.999%	>99.999%	>99.999%	100%
**Fixed-period heartbeat monitoring**	0	0	0	0	0	100%
